# Homogentisate 1,2-dioxygenase (*HGD*) gene variants in young Egyptian patients with alkaptonuria

**DOI:** 10.1038/s41598-023-41200-7

**Published:** 2023-09-01

**Authors:** Zeinab S. Abdelkhalek, Iman G. Mahmoud, Heba Omair, Mohamed Abdulhay, Mohamed A. Elmonem

**Affiliations:** 1 Clinical and Chemical Pathology Department, Faculty of Medicine, Cairo University, Center of Social and Preventive Medicine, Room 409, Monira, Cairo, 11628 Egypt; 2 Metabolic Division, Pediatrics Neurology Department, Faculty of Medicine, Cairo University Children’s Hospital, Cairo, Egypt; 3 Pediatrics Department, Faculty of Medicine, Helwan University, Cairo, Egypt

**Keywords:** Genotype, Medical genetics, Mutation, Sequencing, Molecular medicine

## Abstract

Alkaptonuria (AKU) is a rare autosomal recessive metabolic disorder caused by pathogenic variants in the homogentisate 1,2-dioxygenase (*HGD)* gene. This leads to a deficient HGD enzyme with the consequent accumulation of homogentisic acid (HGA) in different tissues causing complications in various organs, particularly in joints, heart valves and kidneys. The genetic basis of AKU in Egypt is completely unknown. We evaluated the clinical and genetic spectrum of six pediatric and adolescents AKU patients from four unrelated Egyptian families. All probands had a high level of HGA in urine by qualitative GC/MS before genetic confirmation by Sanger sequencing. Recruited AKU patients were four females and two males (median age 13 years). We identified four different pathogenic missense variants within *HGD* gene. Detected variants included a novel variant c.1079G > T;p.(Gly360Val) and three recurrent variants; c.1078G > C;p.(Gly360Arg), c.808G > A;p.(Gly270Arg) and c.473C > T;p.(Pro158Leu). All identified variants were properly segregating in the four families consistent with autosomal recessive inheritance. In this study, we reported the phenotypic and genotypic spectrum of alkaptonuria for the first time in Egypt. We further enriched the *HGD*-variant database with another novel pathogenic variant. The recent availability of nitisinone may promote the need for genetic confirmation at younger ages to start therapy earlier and prevent serious complications.

## Introduction

Alkaptonuria (AKU, OMIM# 203500) is a rare autosomal recessive inborn error of metabolism. It is caused by pathogenic variants in the homogentisate 1,2-dioxygenase gene (*HGD*, OMIM* 607474), commonly causing a complete deficiency of the HGD enzyme (EC 1.13.11.5), with the consequent accumulation of homogentisic acid (HGA) derived from the metabolism of the amino-acids tyrosine and phenylalanine^1^. HGA in alkaptonuria is commonly excreted in massive amounts in urine, causing its darkening upon air exposure, which is considered a pathognomonic sign of the disease. Furthermore, HGA is also deposited as an “ochronotic” pigment when oxidized within the articular connective tissues causing ochronotic arthritis, a degenerative joint disease^1,2^. Extra-articular complications of the disease include cardiovascular manifestations, such as mitral and aortic valve calcification with stenosis or regurgitation, renal and prostatic stones and hypothyroidism^3^.

The *HGD* gene is located on the long arm of chromosome-3 (3q13.33) and contains 14 exons. The HGD protein consists of 445 amino acids, with a protein expression pattern mainly localized to the liver and kidney, but also expressed in other organs, such as gallbladder, gastrointestinal tract, prostate, thyroid and cartilage (https://www.proteinatlas.org/ENSG00000113924-HGD/tissue)^4^. Darkening of urine upon standing is the earliest sign of alkaptonuria and in our experience can be noticed immediately after birth in some cases. It is commonly the main clinical feature of the disease in pediatric patients and many affected individuals may remain clinically asymptomatic until the third or fourth decades of life^5^.

When AKU is suspected, the presence of homogentisic acid in urine can be assayed by gas chromatography mass spectrometry (GC/MS), which serves as a quick diagnostic marker^6^. However, being a monogenic disease with a relatively short gene, Sanger sequencing of the 14 exons of *HGD* with their exon/intron boundaries is most convenient and at the same time critical to confirm affected individuals and carriers of the disease^1^.

The worldwide incidence of alkaptonuria is approximately 1:250,000–1,000,000 with relative hot spots in Slovakia, the Dominican Republic, India and Jordan^7,8^. The actual prevalence of AKU may be underestimated due to the subtle signs of the disease, causing many patients to go undiagnosed for most of their lives^1,8^. Although the prevalence of AKU in Arab countries is not well studied, it is likely to be higher than in most populations due to the autosomal recessive nature of the disease and the high prevalence of consanguineous marriages in the region^9^. No data about the disease incidence or genotype in Egyptian AKU patients is yet available.

This study was conducted to investigate the genetic makeup of Egyptian patients with suspected alkaptonuria based on clinical manifestations and elevated HGA in urine. We performed Sanger sequencing of the *HGD* gene in all suspected individuals with their parents and available siblings in a trial to confirm pathogenicity of the detected variants and capture presymptomatic cases and carriers in siblings of affected individuals.

## Patients and methods

### Patients

We recruited four unrelated Egyptian families with suspected AKU from different locations within Egypt. Probands and their affected siblings were followed up at the metabolic clinic at Cairo University Children's Hospital over a duration ranging from 1 to 16 years. We sampled a total of 17 individuals from the four families. AKU patients were recruited based on family history, clinical examination and the presence of homogentisic acid in urine using a qualitative GC/MS technique. The urinary organic acid analysis was performed by the Agilent 7890B GC with Agilent 7010 triple quadropole GC/MS and 7693A autosampler (Agilent Technologies, Santa Clara, CA, USA) using non-labelled internal standards and following the methodology of La Marca and Rizzo^10^. Written informed consent was obtained from all participants prior to inclusion in the study. The study was conducted in accordance with the declaration of Helsinki for studies involving human participants and the study protocol was approved by the Research Ethics Committee at Faculty of Medicine, Cairo University, Egypt (Approval code #N-71–2022).

### Genomic DNA extraction and *HGD* sanger sequencing

EDTA blood was collected from all suspected AKU patients, their parents and all available healthy siblings. Genomic DNA was extracted using QIAamp® DNA Blood Mini Kit (QIAGEN, Germany). The quantity and quality of the extracted DNA were evaluated by the NanoDrop2000® (Thermo Scientific, USA) and DNA was stored at − 20 °C until further processing.

The *HGD* gene of each index case was sequenced using specific primers designed with the help of Integrated DNA Technologies, OligoAnalyzer™ Tool (https://www.idtdna.com/calc/analyzer). The sequences of all used primers are listed in Supplementary Table 1. This was followed by family segregation analysis for the detected variant in each proband in the corresponding exon for both parents and all available siblings. PCR reactions were conducted using Dream-Taq Green PCR Master-Mix (Thermo Scientific, USA). In short, an initial denaturation for 10 min at 95 °C was followed by 35 cycles of (30 s denaturation at 95 °C, 45 s annealing at 56–64.6 °C and extension for 1 min at 72 °C). Final extension at 72 °C was for 10 min. The specific annealing temperatures for different primer pairs are provided in Supplementary Table 1. PCR products were run by electrophoresis using 2% agarose gel to confirm band specificity. DNA was then purified from the amplification reaction with the GeneJET™ Genomic DNA Purification Kit (Applied Biosystems, USA) according to manufacturer’s protocol. Purified DNA was stored at − 20 °C until sequenced using the Brillant Dye™ terminator V3.1 Cycle Sequencing kit (NimaGen, the Netherlands) through the ABI 3500 gene analyzer.

## Results

### Clinical features

**Figure 1:**
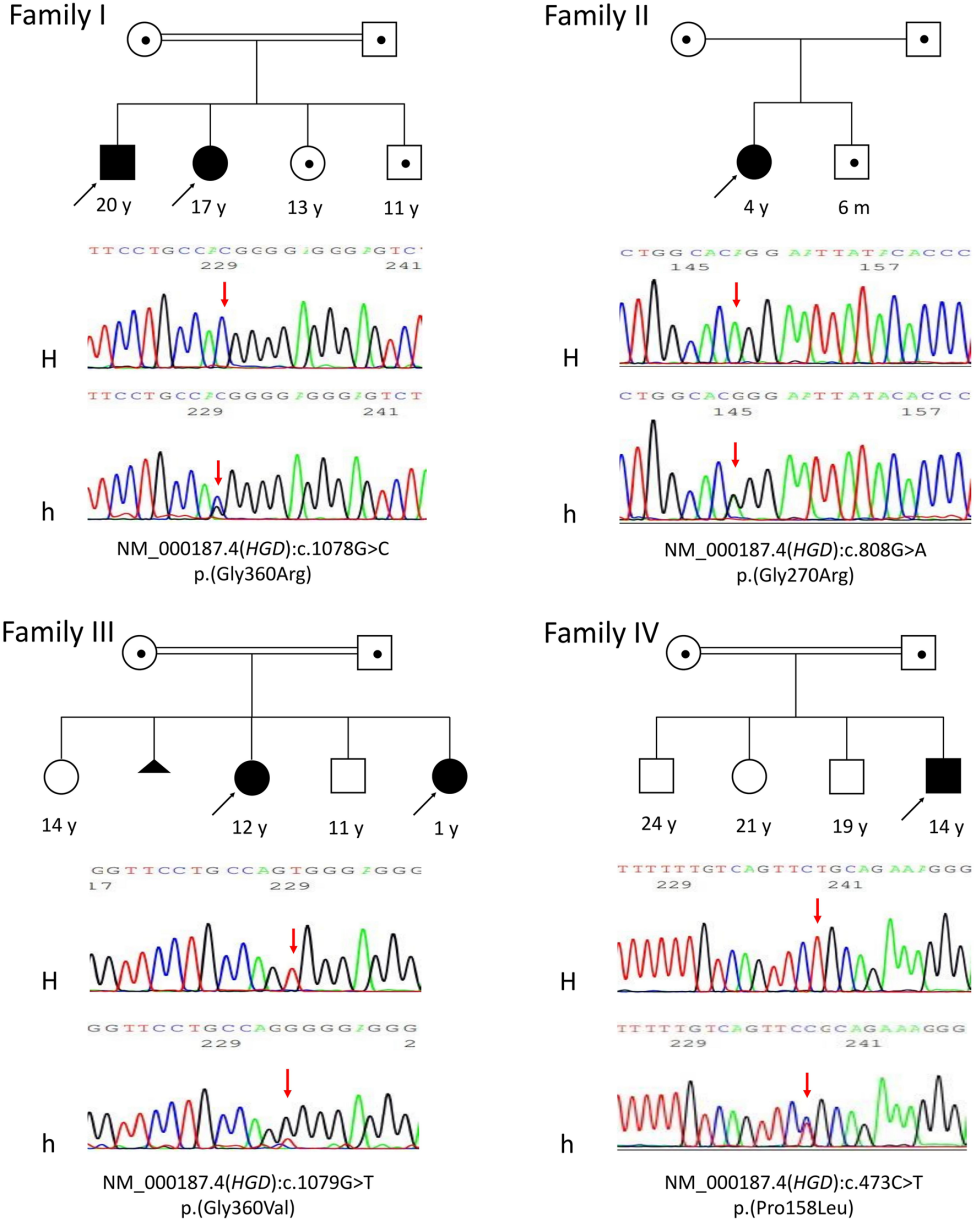
Family pedigrees and genetic variants of affected AKU patients. All detected variants were fully segregated in all tested family members (homozygous in the AKU patients, heterozygous in parents and heterozygous or wild type in unaffected siblings) consistent with the autosomal recessive inheritance nature of the disease. H, Homozygous; h, heterozygous. In family pedigrees homozygous individuals are marked in black, while confirmed heterozygous carriers are marked with a black dot inside.

Six AKU patients from four unrelated Egyptian families were recruited in the current study (4 females/2 males, 1–20 years). Three families were reported consanguineous (parents were first cousins), while in family II, consanguinity was denied; however, both parents originated from the same small village and a distant common ancestor cannot be excluded. Demographic and clinical features of affected individuals are summarized in Table 1, while family pedigrees are provided in Fig. 1.

**Table 1:** Demographic and clinical features of AKU patients (n = 6).

Family/patient no	Gender	Current age (years)	Age at diagnosis (months)	Consanguinity	Clinical manifestations					Relevant investigations
					Renal/urinary	Skin, connective tissue and joints	Ocular and auditory	Cardiac	Others	
I/1	Male	20	72	1st cousins	Darkening of urine upon standing(first sign)	Arthralgia of weight bearing joints, pain of lower limbs	–	–	Mental subnormality	IQ = 60, CT abdomen: Both kidneys are seen on the left side
I/2	Female	17	36	1st cousins	Darkening of urine upon standing(first sign)	Arthralgia of weight bearing joints, pain in both arms, low back and lower limbs, short stature	High myopia, strabismus, deafness	Rheumatic heart disease and receives long acting penicillin	Mental subnormality	IQ = 70, ABR: bilateral severe to profound sensorineural hearing loss, fundus; High myopic, normal electroretinogram (ERG), normal karyotype, mild mitral regurge in transthoracic echocardiography, increased serum ASOT and ESR, urine urate crystals + + + , delayed bone age by 2 years
II/3	Female	4	6	Not reported but parents originate from same village	Pink urine in diapers, darkened upon standing (first sign), urinary tract infections	Hyper-pigmentation on back with brown discoloration of buttocks	–	–	–	–
III/4	Female	12	6	1st cousins	Reddish pink urine in diapers with darkening upon standing (first sign)	Brownish pigmentation of buttocks and a bluish patch on the arm, arthralgia, dental caries	Mild periorbital edema	–	–	Increased calcium/creatinine ratio, micro-hemoglobinuria, elevated liver enzymes almost double, normal abdomino-pelvic CT with contrast
III/5	Female	1	1	1st cousins	Pinkish urine in diapers with darkening upon standing (only manifestation)	–	–	–	–	–
IV/6	Male	14	72	1st cousins	Pink urine in diapers which darkens upon standing (first sign)	Black ear cerumen, arthralgia of weight bearing joints, dental caries, mild periorbital skin darkening	–	Chest pain on exercise. rheumatic heart excluded, symptoms improved on decreasing protein intake	–	Mild left ventricular and septal hypertrophy on echocardiography, mild hypertrophic cardiomyopathy with normal systolic function, increased ASOT, paroxysmal atrial tachycardia with sinus rhythm on ECG

The proband of family I (Patient I-1) is currently a 20-year-old male and his sister (I-2) is 17 years old. The family was from Beni-Suef governorate in Central Egypt (Supplementary Fig. 1). Both affected siblings had mild intellectual disability and complained of intractable pains in weight bearing joints starting in mid-adolescence (12–14 years). The older male sibling had congenital anomaly in the kidney, while his sister had severe sensorineural hearing loss, high myopia, strabismus, delayed bone age, short stature and mitral regurgitation (Table 1). The latter could be attributed to rheumatic heart disease as she was receiving long acting penicillin and had persistently increased ESR and ASOT titers.

The other two siblings, although having a normal organic acid profile in urine and only being carriers for the pathogenic variant detected in homozygosity in their AKU siblings, they were also suffering. The third female sibling had bilateral severe to profound sensorineural hearing loss and the fourth male sibling had mild hepatosplenomegaly and right iliac bone lesion that was initially suspected as an alkaptonuric joint lesion before alkaptonuria being excluded by urinary organic acid and genetic analyses.

Family II had two siblings, a female (proband) and an unaffected male. The proband (II-3) was diagnosed with AKU through HGA detection in urine when she was 6-month-old. Her only manifestations so far are darkening of urine upon standing and hyper pigmentation and brown discoloration of buttocks (Table 1). Her sibling was symptom free and was revealed as heterozygous by genetic analysis.

Family III consisted of four siblings, three females including two AKU patients and one male sibling. The proband (III-4) was diagnosed with AKU at 6 months of age. She recently developed hyperpigmentation of buttocks and a long-standing bluish-brownish patch on the arm. Consequently, due to the experience gained by the mother, her sister (III-5) was spotted at one month of age with the same blackish discoloration of urine (Table 1). The other two siblings were completely healthy.

The proband (IV-6) of family IV was the youngest male and the only affected member among three older siblings. He had normal motor and mental development. Parents noticed pink to light red urine and black ear cerumen immediately after birth. At the age of 2 months, he was misdiagnosed as having urinary bleeding, although all his renal functions were normal. At the age of 6 years he started having joint pains and recurrent abdominal pain (Table 1). Alkaptonuria was then suspected and was confirmed by GC/MS. All six AKU patients were confirmed to have massive amounts of homogentisic acid in urine by qualitative organic acid analysis by GC/MS.

### Genetic diagnosis

Sanger sequencing of the *HGD* gene revealed the existence of four different missense pathogenic/likely pathogenic variants in exons 8, 11 and 13 in the four recruited families including a novel pathogenic variant in exon 13 detected in family III (Table 2).

**Table 2:** *HGD* pathogenic variants detected in Egyptian AKU families.

Family	Patients	Chromosomal location (GRCh38)	Nucleotide change* (Zygosity)	Exon	Protein effect*	Variant effect	dbSNP ID	ACMG classification	Clinivar prediction	MAF exomes/genomes in gnomAD	References
I	1, 2	Chr3-120633257-C-G	c.1078G > C (H)	13	p.(Gly360Arg)	Missense	rs368717991	Pathogenic	Pathogenic	0.0000119/0.0000131	Porfirio et al.^12^
II	3	Chr3-120641660-C-T	c.808G > A (H)	11	p.(Gly270Arg)	Missense	rs120074174	Pathogenic	Pathogenic	0.0000557/0.0000526	Muller et al.^13^
III	4, 5	Chr3-120633256-C-A	c.1079G > T (H)	13	p.(Gly360Val)	Missense	rs1172885188	Pathogenic	–	0.0/0.00000658	This study
IV	6	Chr3-120647049-G-A	c.473C > T (H)	8	p.(Pro158Leu)	Missense	rs375396766	Likely pathogenic	Uncertain significance	0.0000239/0.00000657	Vilboux et al.^14^

*Variants nomenclature is according to *HGD*: NM_000187.4 and NP_000178.2 Refseq gene transcript and protein, respectively; MAF, minor allele frequency according to gnomAD population database (https://gnomad.broadinstitute.org/); H, homozygous. All variants segregated properly in both parents and available siblings of the diseased children according to strict autosomal recessive inheritance.

All detected variants fully segregated in all tested family members (homozygous in the AKU patients, heterozygous in parents and heterozygous or wild type in available unaffected siblings) consistent with the autosomal recessive inheritance nature of the disease (Fig. 1).

The first family had a missense pathogenic variant in *HGD* exon 13; NM_000187.4:c.1078G > C, NP_000178.2:p.(Gly360Arg). The variant is recurrent and has been reported previously in the *HGD* variant database and predicted to be pathogenic according to the American College of Medical Genetics (ACMG) criteria for the classification of pathogenic variants^11^ (Table 2). The variant has been previously reported as disease causing in several studies^12,15^.

Family II also had a missense pathogenic variant in *HGD* exon 11; NM_000187.4:c.808G > A, NP_000178.2:p.(Gly270Arg). It is also predicted as pathogenic according to ACMG and ClinVar (Table 2). The variant has been previously reported as disease causing in several studies^13–16^.

Family III had a novel missense pathogenic variant in exon 13 of *HGD* gene; NM_000187.4:c.1079G > T, NP_000178.2:p.(Gly360Val). This novel variant was detected in homozygousity in the proband (III-4) and her affected sister (III-5), while it was heterozygous in their parents. It is predicted to be pathogenic based on the ACMG criteria PM1, PM5, PM2, and PP3^11^. The variant belongs to a hot-spot region of 17 amino-acids in the HGD enzyme, which has 13 missense/in-frame variants including 11 pathogenic variants, 1 uncertain variant and 1 benign variant, thus qualifying as pathogenic strong (PM1). Three alternative variants at the same amino-acid location are also reported pathogenic in ClinVar (c.1078G > a; p.(Gly360Arg), c.1078G > C; p.(Gly360Arg) and c.1079G > C; p.(Gly360Ser)) (PM5). Furthermore, it is extremely rare in population databases (Minor allele frequency in gnomAD exomes: 0.00 and in gnomAD genomes: 0.00000658) (PM2) and the absolute majority of predictor tools evaluate the variant as pathogenic (PP3).

The fourth family (IV) had a missense likely pathogenic variant in exon 8 of *HGD* gene NM_000187.4:c.473C > T; NP_000178.2:p.(Pro158Leu). This variant was detected in homozygous state in the proband (IV-6) and heterozygous in both parents. It is predicted to be likely pathogenic according to ACMG criteria^11^; however, it was reported as variant of uncertain significance in ClinVar (Table 2). The variant has been also previously reported as disease causing^14,17^.

## Discussion

In the current study, we evaluated the clinical and genetic characteristics of four unrelated Egyptian families suffering from alkaptonuria. All patients presented with isolated dark urine at a median age of 6 months and were confirmed to have a high level of homogentisic acid in urine by qualitative GC/MS evaluation of organic acids in urine.

AKU is a rare metabolic disorder that is almost forgotten in Egypt and in the Arab region, as only a few cases were reported from Jordan^18^, Algeria^19^, UAE^20^, Iraq^21^, Lebanon^22^ and Morocco^23^. Furthermore, the prevalence and genetic makeup of the disease are completely unknown in Egypt, although expected to be more prevalent than developed countries due to higher rates of consanguineous marriages that propagate the autosomal recessive inheritance of the disease. We conducted this study to identify the underlying *HGD* pathogenic variants within Egypt causing AKU and to raise awareness among Egyptian health care professionals about the disease. Furthermore, we wanted to establish the routine genetic confirmation of AKU in Egypt before the authorization of the specific therapeutic agent, nitisinone in Egypt.

In our study, we identified four different variants causing AKU, including three recurrent variants reported in several populations as disease causing: c.1078G > C; p.(Gly360Arg) detected in family I, has been reported in patients from Italy, Australia, UK, France, Spain, India and the USA. The variant c.808G > A; p.(Gly270Arg) detected in family II, has been previously reported in patients from Italy, Slovakia, France, Armenia, Brazil, USA, Dominican republic, Turkey, the UK, Russia, Germany, Peru and India, while c.473C > T; p.(Pro158Leu) detected in family IV in our study, has been previously reported in patients from USA, Canada, Macedonia, and Taiwan^15^.

The last detected variant in our study is a novel missense pathogenic variant c.1079G > T; p.(Gly360Val) that was encountered in family III. This variant is not reported in ClinVar; however, it lies within a mutational hotspot in exon 13 of the *HGD* gene, which contains over ten missense pathogenic variants including the detected variant at the same amino acid location in family I, c.1078G > C;p.(Gly360Arg), indicating that this domain of the HGD protein is very sensitive to amino-acid alterations, and non-synonymous missense variants most likely will result in a deleterious effect on the protein function. All four variants were detected in homozygous state in affected patients and segregated properly in their families.

Although many of our patients had arthralgic pain in weight bearing joints, especially adolescents, they did not have evident radiographic abnormalities commonly detected in adult AKU patients, such as spine osteoporosis, calcification of the nucleus pulposus, osteophytosis or reactive sclerosis of the articular surfaces^24^. They also didn't have any noticeable teeth discoloration characteristic of alkaptonuria^25^, and repeated clinical examination didn't pick up any cardiac or renal complications, which is mainly related to the relatively young age of most of our patients.

The recent availability of the new medication nitisinone (2-(2-nitro-4-trifluoromethylbenzoyl)-1,3-cyclohexanedioneor, NTBC), which inhibits the enzyme 4-OH phenylpyruvate dioxygenase, thus blocking the formation of HGA, enhances the need for genetic confirmation and carrier frequency detection in families before starting this rather expensive therapy.

Low dose nitisinone in the range of 0.2–2 mg daily reduces urinary HGA excretion by > 90%, which may prevent AKU-related complications in the long run^26^. This drug has been used for more than 20 years for the treatment of children with tyrosinemia type I^27^. An earlier start of nitisinone together with a diet restricting phenylalanine and tyrosine intake may lead to an improved long-term functional outcome. At the same time, nitisinone treatment should be cautiously monitored to avoid the development of mental and cognitive disturbances^28–30^. The low dose used should also reduce the possibility of increased plasma tyrosine levels, which may cause nitisinone keratopathy. In a recent study conducted by Ranganath et al., to compare the effects of nitisinone 2 mg and 10 mg in the treatment of alkaptonuria, they reported that nitisinone 10 mg was more efficient in slowing the disease progression; however, it simultaneously increased tyrosine levels and the incidence of corneal keratopathy (14.5% vs 4.9%)^31^. Furthermore, ntisinone is still a very expensive drug, so one of the major advantages of the low-dose therapy will be its economic benefit, especially in a resource-limited country, such as Egypt.

The early development of remarkable and progressive joint complaints in the majority of pediatric and adolescent Egyptian AKU patients in our study indicates that diet restriction of phenylalanine and tyrosine and supportive therapeutic strategies are not efficient in controlling HGA deposition in tissues and that nitisinone, which is not yet covered by insurance in Egypt, is needed.

Identifying the underlying genetic variants of families with AKU in Egypt should have an impact on health community policy towards the disease. Since heterozygous variants have a major role as one of the liability genetic determinants in subsequent generations, raising awareness regarding premarital genetic counseling at least in affected families may break the line of inheritance in such disorders. Furthermore, biochemical analysis of HGA in urine and genetic confirmation should be routine screening steps for all family members of affected individuals to detect presymptomatic cases, especially that a specific therapy that can minimize long term complications is currently available.

Concerning family I, we further recommend whole exome or genome sequencing, as their complex genetic trait requires a more extensive genetic sequencing technique to fully elucidate the etiology behind their apparently syndromic intellectual disability and sensorineural hearing loss.

In conclusion, in this study we explored the phenotypic and genotypic spectrum for alkaptonuria for the first time in Egypt. Although we revealed the genetic makeup of only a limited number of Egyptian patients, this study may form a base to be built upon for establishing clinical genetic screening and family counseling of AKU in Egypt and surrounding populations. We further enriched the mutational spectrum of the *HGD* gene with a novel pathogenic variant detected in two AKU siblings.

### Supplementary Information

Supplementary Information.

## Data Availability

All relevant data generated or analyzed during this study are included in this published article and supplementary materials. Any further clinical details are available from the corresponding author on reasonable request. Novel *HGD* variant c.1079G > T; p.(Gly360Val) detected in family 3 has been submitted to the Leiden Open Variation Database (LOVD) under variant ID: #0000918409.
