# Supplementary material for: Homogentisate 1,2-dioxygenase (HGD) gene variants in young Egyptian patients with alkaptonuria
Source: Sci Rep. 2023 Sep 1;13:14374. doi: 10.1038/s41598-023-41200-7 (PMC10474279; doi:10.1038/s41598-023-41200-7)
Supplement: Supplementary file 1 — Supplementary Information. [file 41598_2023_41200_MOESM1_ESM.docx]

**Homogentisate 1,2-dioxygenase (*HGD*) gene variants in young Egyptian patients with alkaptonuria**

**Zeinab S. Abdelkhalek^1,^ *, Iman G. Mahmoud^2^, Heba Omair^1^, Mohamed Abdulhay^3^, Mohamed A. Elmonem^1^**

^1^ Clinical and Chemical Pathology Department, Faculty of Medicine, Cairo University, Cairo, Egypt

^2^ Pediatrics Neurology Department, Metabolic Division, Faculty of Medicine, Cairo University Children’s Hospital, Cairo, Egypt

^3^ Pediatrics Department, Faculty of Medicine, Helwan University, Cairo, Egypt

Supplementary Table1: PCR primers for genomic amplification of the human *HGD* exons

(5’ → 3’).

| **Primer location** | **Primer Name** | **Primer sequence** | **Number of bases** | **Annealing temperture** | **Amplicon size (bp)** |
| --- | --- | --- | --- | --- | --- |
| **Exon 1** | HGD_EX1_F | CCCTGTGTATGAGTTAGACA | 20 | 56 | 294 |
|  | HGD_EX1_R | CTCTAAGTCTTTTCCAACTCTG | 22 |  |  |
|  |  |  |  |  |  |
| **Exon2** | HGD_EX2_F | GCAATATCCAGCACTCTTCTGA | 22 | 57.7 | 437 |
|  | HGD_EX2_R | CCCCTATGACTTGGGAAACC | 20 |  |  |
|  |  |  |  |  |  |
| **Exon3** | HGD_EX3_F | GGGGCAAGTCACATCAAAAG | 20 | 64.6 | 415 |
|  | HGD_EX3_R | GCTGGCAGGAAGTTCATTCT | 20 |  |  |
|  |  |  |  |  |  |
| **Exon4** | HGD_EX4_F | TTGGCAGCATGGAAATAACC | 20 | 56.6 | 514 |
|  | HGD_EX4_R | TGAGCAGAAAACAGACACACT | 21 |  |  |
|  |  |  |  |  |  |
| **Exon5** | HGD_EX5_F | AAAACTCACTGATGTGCCTGG | 21 | 58.5 | 526 |
|  | HGD_EX5_R | TTGTCTCTGCTGCCTCCT | 18 |  |  |
|  |  |  |  |  |  |
| **Exon6** | HGD_EX6_F | ATGAGTCAGTAAATTCAGGCTC | 22 | 64.6 | 471 |
|  | HGD_EX6_R | GATGTTCAGTCAAGATAAATGCC | 23 |  |  |
|  |  |  |  |  |  |
| **Exon7** | HGD_EX7_F | GTCGCTATTCTTTCATTCCCTC | 22 | 64 | 532 |
|  | HGD_EX7_R | GTCCAGAAGAGATGGGCAAA | 20 |  |  |
|  |  |  |  |  |  |
| **Exon8** | HGD_EX8_F | CCCCTTTTTGACCATGTCAC | 20 | 61 | 473 |
|  | HGD_EX8_R | CTCAGATTCCCTCCTCGTTG | 20 |  |  |
|  |  |  |  |  |  |
| **Exon9** | HGD_EX9_F | CATGTTTGCTCTGGTCACCT | 20 | 64 | 400 |
|  | HGD_EX9_R | TTTTCTGGGAAGACACTTGGTC | 22 |  |  |
|  |  |  |  |  |  |
| **Exon10** | HGD_EX10_F | CTCTCTTCCCTTCCCCTCAC | 20 | 61 | 551 |
|  | HGD_EX10_R | TTTGTAGTGCCGTAGTGGTATGA | 23 |  |  |
|  |  |  |  |  |  |
| **Exon11** | HGD_EX11_F | ATACTTCTCCCAAAGGACGG | 20 | 63.1 | 396 |
|  | HGD_EX11_R | CTCCCTCACCAAAGGACAAA | 20 |  |  |
|  |  |  |  |  |  |
| **Exon12** | HGD_EX12_F | CCCTACCCCAAACCTCAGTA | 20 | 58.2 | 595 |
|  | HGD_EX12_R | CACGAGCCAAATGAACCTCT | 20 |  |  |
|  |  |  |  |  |  |
| **Exon13** | HGD_EX13_F | TGCCAAGAATGCCAATATGA | 20 | 61 | 477 |
|  | HGD_EX13_R | CCTCTTTTGACTCTTCCTCTG | 21 |  |  |
|  |  |  |  |  |  |
| **Exon14** | HGD_EX14_F | TTGTACCAGGTCTCACCAG | 19 | 58.7 | 610 |
|  | HGD_EX14_R | CTGCCAGGTTTGTCTCATCA | 20 |  |  |


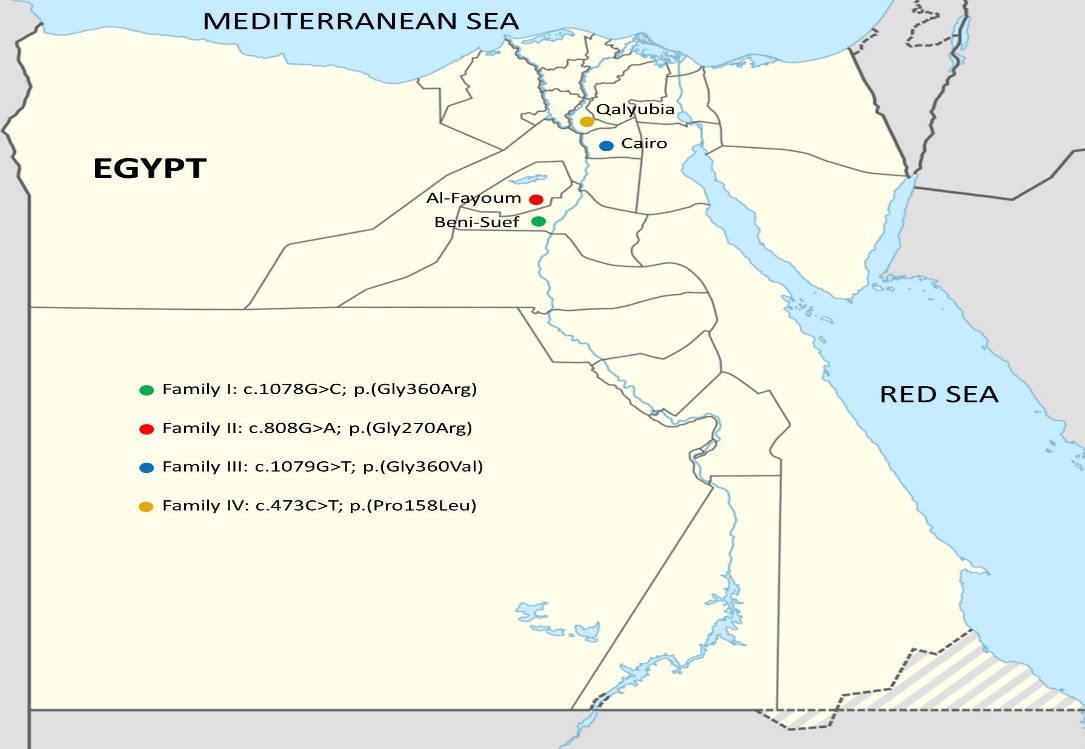


Supplementary Figure 1: Demographic location of reported AKU families within Egypt.
